# Environmental *Streptococcus uberis* Associated with Clinical Mastitis in Dairy Cows: Virulence Traits, Antimicrobial and Biocide Resistance, and Epidemiological Typing

**DOI:** 10.3390/ani11071849

**Published:** 2021-06-22

**Authors:** Norhan K. Abd El-Aziz, Ahmed M. Ammar, Hend M. El Damaty, Rehab A. Abd Elkader, Hosam A. Saad, Waleed El-Kazzaz, Eman Khalifa

**Affiliations:** 1 Department of Microbiology, Faculty of Veterinary Medicine, Zagazig University, Zagazig 44511, Egypt; prof.ahmedammar_2000@yahoo.com; 2 Department of Animal Medicine, Infectious Diseases, Faculty of Veterinary Medicine, Zagazig University, Zagazig 44511, Egypt; 3 Belbies Veterinary Organization, Ministry of Agriculture, Belbies 44974, Egypt; alrehablab01@gmail.com; 4 Department of Chemistry, College of Science, Taif University, P.O. Box 11099, Taif 21944, Saudi Arabia; h.saad@tu.edu.sa; 5 Molecular Microbiology Lab., Botany and Microbiology Department, Faculty of Science, Suez Canal University, Ismailia 41522, Egypt; walid_elkazaz@science.suez.edu.eg; 6 Department of Microbiology, Faculty of Veterinary Medicine, Matrouh University, Matrouh 51511, Egypt; khalifa.eman@alexu.edu.eg

**Keywords:** antimicrobial resistance, biocides, dairy cows, intramammary infections, RFLP–PCR, *Streptococcus uberis*, virulence

## Abstract

**Simple Summary:**

Mastitis remains one of dairy cattle’s most perplexing and expensive diseases. This study is the first to look into the virulence traits, antimicrobial and biocide resistance, and epidemiological typing of *Streptococcus uberis* (*S. uberis*) isolated from bovine clinical mastitis in dairy farms of diverse hygienic interventions in Egypt. The overall *S. uberis* infection rate was 20.59%; all were multidrug-resistant (MDR). The *sua* gene was the most frequent virulence gene (42.02%), followed by *pau*A (40.57%), *cfu* (21.73%), *skc* (20.28%), and *opp* (11.59%). The *erm*(B) gene serves as the predominant antimicrobial-resistant gene (75.36%), followed by *fexA* (52.63%) and *tet*(M), *blaZ*, and *aac(6′)aph(2″)* genes (46.38% each). Of note, 79.71% of *S. uberis* isolates carried *qac* genes; among them, 55 (79.71%), 54 (78.26%), and 13 (18.84%) harbored *qacED1*, *qacC/D*, and *qacA/B* genes, respectively. Restriction fragment length polymorphism–polymerase chain reaction (RFLP–PCR) indicated that all analyzed isolates were *S. uberis* type I by their unique RFLP pattern. This study shows a significant variation in the occurrence of virulent *S. uberis* in dairy cows with clinical mastitis regarding the prospective hygienic concerns. Furthermore, MDR coupled with the existence of biocide resistance genes indicates the importance of *S. uberis* surveillance and the prudent use of antimicrobials in veterinary clinical medicine to avoid the dissemination of antimicrobial resistance.

**Abstract:**

Mastitis remains a serious problem for dairy animals. The misappropriation of antimicrobial agents helps accelerate resistance, which poses a serious challenge in controlling environmental *S. uberis* infection. Here, we study the virulence attributes, antimicrobial and biocide resistance, and epidemiological typing of *S. uberis* recovered from bovine clinical mastitis in dairy farms of diverse hygienic interventions in Egypt. The overall *S. uberis* infection rate was 20.59%; all were multidrug-resistant (MDR). The *sua* gene was the most frequent virulence gene (42.02%), followed by *pau*A (40.57%), *cfu* (21.73%), *skc* (20.28%), and *opp* (11.59%). The *erm*(B) gene served as the predominant antimicrobial-resistant gene (75.36%), followed by *fexA* (52.63%) and *tet*(M), *blaZ*, and *aac(6′)aph(2″)* genes (46.38% each). Of note, 79.71%, 78.26%, and 18.84% of *S. uberis* isolates harbored *qacED1*, *qacC/D*, and *qacA/B* genes, respectively. All analyzed isolates were *S. uberis* type I by their unique RFLP–PCR pattern. In conclusion, the sustained presence of *pauA* and *sua* genes throughout the investigated farms contributes to a better understanding of the bacterium’s pathogenicity. Furthermore, MDR coupled with the existence of biocide resistance genes indicates the importance of *S. uberis* surveillance and the prudent use of antimicrobials in veterinary clinical medicine to avoid the dissemination of antimicrobial resistance.

## 1. Introduction

Mastitis is a significant concern affecting dairy animals worldwide, causing great losses to breeders and impacting the country’s national income [1,2]. Environmental streptococci, notably *Streptococcus uberis* (*S. uberis*), are among the main contributing agents of mastitis in many countries and have increased their significance for udder health in recent decades [3]. This pathogen is not obligatorily adapted to the udder but is ubiquitous as it is considered an environment-associated straw bedding and pasture pathogen [4]. Since *S. uberis* is the prime pathogen in a dairy herd, frequent antimicrobial treatments and several environmental factors favor the development of this form of mastitis [3].

*Streptococcus uberis* has previously been categorized into two distinct types, I and II; both were isolated from bovine mastitis cases, the latter being reclassified as *Streptococcus parauberis* (*S. parauberis*) [5]. It is impossible to differentiate between *S. uberis* and *S. parauberis* using phenotypic methods [6]. However, *S. uberis* isolates were verified by 16S rRNA gene restriction fragment length polymorphism (RFLP) using the *Hha*I restriction endonuclease for further identification of the *S. uberis* genetic variation [7]. 

Despite the economic effect of the high prevalence of environmental streptococci in dairy herds, virulence factors related to the pathogenicity of *S. uberis* are not well characterized; these comprise a significant existential threat to the implementation of control strategies [8]. Various potentially virulence factors were identified for *S. uberis*, among these, *sua*, *cfu*, *opp*, *skc*, and *pauA*, that play prominent roles in the adherence and early colonization of bovine mammary epithelial cells [9,10,11]. 

Antimicrobial resistance is one of the world’s leading threats to human and animal health [12]. It appears to have an extreme occurrence among streptococcal isolates of mastitis in Egypt [13] and *S. uberis* in many countries [4,14,15]. However, this susceptibility can vary from one region to another. Even within the same region, it is necessary to monitor the pathogens’ resistance to the antimicrobials used in the treatment of mastitis in various areas [16]. In Egypt, most bovine mastitis studies have focused on the inclusion of *Escherichia coli*, *Staphylococcus aureus*, *Streptococcus agalactiae*, and, infrequently, *S. uberis* [13,17,18]. 

Disinfectants based on quaternary ammonium compounds (QACs) have a wide range of veterinary medicine implementations and are critical in controlling animal diseases. They are widely used worldwide, which can contribute to bacterial resistance [19]. 

In Egypt, there has been no exploration of the existence of *S. uberis*-associated virulence genes in mastitic dairy cows and the plausible allocation of virulence dynamics in the distinct hygiene measures applied in dairy farms. Moreover, there are few studies on *S. uberis* resistance to antimicrobials as well as biocides. Therefore, this study was designed to explore the following points: (i) ascertaining the infection rate of *S. uberis* in dairy cows and the hygiene correlation with its abundance, (ii) detecting the most prospective virulence-associated, antimicrobial and antiseptic resistance genes in environmental *S. uberis* isolates using conventional PCR, and (iii) determining the genotypic variation among the virulent *S. uberis* isolates using RFLP–PCR.

## 2. Materials and Methods

### 2.1. Lactating Cows and Husbandry Practices

The lactating cows under study were chosen from dairy farms of three distinct hygienic interventions in Alexandria (A) and Sharkia Governorates (B), as well as some individual smallholder cases in different villages of Sharkia Governorates (C and D), over a year, from July 2017 to August 2018. The udder of each lactating cow was screened for recurrent clinical mastitis. Hygienic interventions were based entirely on the following criteria: (i) periodic monitoring of mastitis by an indirect field check during the lactation season, such as the California mastitis test, (ii) pre-milking procedures, such as udder washing and pre- and post-milking teat dipping with antimicrobial dip; and (iii) dry period treatment after the last lactation, bedding materials, and environmental hygiene as well as balanced food. In the first farm (A; *n* = 75), lactating cows were milked three times daily through a computerized system using pre- and post-teat dipping. This farm followed the standard routine management, vaccination program, and control measures against infectious diseases with the implementation of all hygienic measures. On the second farm (B; *n* = 50), cows were milked three times daily using a machinery system with post-milking teat dipping and fair, moderate hygienic measures; the cows were placed in straw-bedding barns. The cows of the third farm (C; *n* = 120) and smallholder cows (D; *n* = 90; reared by local farmers in the villages of Sharkia Governorate) were grazed; thus, infection with *S. uberis* from environmental pasture reservoirs was expected. These animals lived in unhygienic environments and were fed on low nutrient rations. The cows were milked twice daily by hand, and there was no disinfection during the milking process.

### 2.2. Milk Sampling and Isolation of S. uberis 

Three hundred and thirty-five milk samples were collected aseptically, just before treatment, from the affected mammary quarters that had clinical signs of abnormal secretions, containing clots or flakes, with udders showing inflammatory symptoms, with or without systemic reaction appearing on the cows. These samples were placed in sterile screw-capped test tubes, kept in an insulated icebox, then transported to the laboratory for further bacteriological and molecular investigations. Bacteriological analysis of milk samples was carried out following conventional protocols [20]. A milk sample loopful was plated onto Edward’s agar medium (Oxoid, Hampshire, UK) and incubated at 37 °C for 24 h. A single, well-isolated colony was subcultured onto a blood agar base (Oxoid, Hampshire, UK) enriched with 7% sterile defibrinated sheep blood and incubated aerobically at 37 °C for 24–48 h. The bacterial isolates were described based on their classic morphological and hemolytic characteristics. Suspected streptococci isolates microscopically appeared as Gram-positive cocci, either in long or short chains. Standard biochemical tests, including catalase, sodium hippurate, and esculin hydrolysis, were carried out [21]. A Christie, Atkins, and Munch-Petersen (CAMP) test was applied [22]. Growth in the presence of 6.5% NaCl at 10 or 45 °C and pH 9.6, combined with resistance to bile salt, was investigated [23]. 

### 2.3. Antimicrobial Susceptibility Testing

The Kirby-Bauer disc diffusion test was used to determine the antimicrobial susceptibilities of *S. uberis* isolates [24]. Commercial discs with the following antimicrobials (Oxoid, Hampshire, England, UK), commonly used in veterinary practices or for public health issues, were selected to perform the antibiogram: penicillin (10 IU), ampicillin (10 µg), amoxicillin (25 μg), amoxicillin–clavulanic acid (20/10 μg), cloxacillin (1 μg), cefoperazone (75 μg), ceftriaxone (30 μg), cephalexin (30 μg), imipenem (10 μg), tetracycline (30 μg), clindamycin (2 μg), erythromycin (15 μg), streptomycin (10 μg), neomycin (30 µg), gentamycin (10 µg), kanamycin (30 µg), novobiocin (30 µg), ciprofloxacin (5 μg), chloramphenicol (30 µg), and trimethoprim–sulphamethoxazole (23.75/1.25 μg). The interpretive criteria used for categorizing an isolate as sensitive or resistant to an antimicrobial agent are established in the Clinical and Laboratory Standards Institute guidelines [25]. Isolates showing resistance to at least three different antimicrobial classes are categorized as multidrug-resistant (MDR) [26]. MAR indices were estimated for each antimicrobial and isolate [27]. 

### 2.4. DNA Extraction and Molecular Identification of S. uberis

*Streptococcus uberis* isolates were cultured in tryptone soya broth (TSB, Oxoid, Hampshire, England, UK) at 37 °C for 24 h. Bacterial DNA was extracted using a QIAamp DNA mini kit (Qiagen GmbH, Hilden, Germany), as recommended by the manufacturer. PCR amplification of the *tuf* gene of *Streptococci* species [28] and the 16S rRNA gene of *S. uberis* [29] was performed using the oligonucleotide primer pairs listed in Table S1 to confirm the conventional bacteriological identification.

### 2.5. PCR Amplifications of Virulence Attributes and Antimicrobial and Biocide Resistance Genes 

Virulence genes for *S. uberis*, *cfu* (encoding for CAMP factor), *opp* (oligopeptide binding protein), *sua* (*S. uberis* adhesion molecule), *pauA* (plasminogen activator), and *skc* (streptokinase activator), were investigated [9,11,30]. The occurrence of antimicrobial resistance genes, conferring resistance to penicillins (*blaZ*), phenicols (*fexA*), aminoglycosides (*aac*(*6′*)*aph*(*2″*)), tetracyclines (*tet*(M), *tet*(O), *tet*(L) and *tet*(K)), macrolides (*erm*(A), *erm*(B) and *erm*(C)), sulfonamide (*sul1)*, and trimethoprim (*dfrA*) was examined [31,32,33,34,35,36,37,38,39,40]. Moreover, PCR targeting *qac* genes, *qacA/B*, *qacC/D*, and *qacED1*, conferring a high level of resistance to antiseptics, was applied [41,42]. Oligonucleotide primer sets and thermal cycling profiles are described in Table S1. The amplification reaction for each gene was conducted with a final volume of 25 μL of the following reaction mixture: 12.5 μL Dream*Taq* Green PCR Master Mix (2X) (Thermo Fisher Scientific, Waltham, MA, USA), 1 μL of each primer (20 pmole), 2 μL template DNA, and 8.5 μL water nuclease-free in a programmable thermal cycler PTC-100 TM (MJ Research Inc., Waltham, MA, USA). *S. uberis* ATCC^®^ 27958™ was used as a reference strain. PCR products were analyzed by agarose gel electrophoresis, stained with ethidium bromide (0.5 µg/mL), and visualized using an ultraviolet transilluminator (Spectroline, Wesbury, Meadville, PA, USA). 

### 2.6. PCR–RFLP 

Epidemiological typing of recovered *S. uberis* isolates was then performed using *Hha*I restriction endonuclease (Thermo Fisher Scientific, Waltham, MA, USA), as described previously [7]. Aliquots of the amplified restriction endonuclease-digested fragments were electrophoresed on 0.5 μg/mL ethidium bromide (Sigma-Aldrich, Chemie GmbH, Schnelldorf, Germany) stained agarose gel with a 100 bp standard DNA molecular weight ladder (Fermentas, Inc., Hanover, NH, USA). The numbers of DNA fragments and their sizes in base pairs were then assessed using Pro-Score/RFLP software version 2.39 (DNA ProScan, Inc.; Nashville, TN, USA). 

### 2.7. Bioinformatics and Statistical Analysis

The overall distribution of the antimicrobial resistance phenotypes, virulence-associated genes, and antimicrobial and biocide resistance genes in *S. uberis* isolates was visualized using a heatmap. The clustering pattern of the isolates and various features were determined by hierarchical clustering dendrogram [43]. These analyses were done using R software (version 3.4.2, R Foundation for Statistical Computing, Vienna, Austria), package pheatmap. To estimate the similarity among *S. uberis* isolates from various farms, the binary distances were calculated among isolates based on the presence or absence of the four studied features (virulence, resistance phenotype/genes, and biocide resistance genes). This analysis was done using the functions *dist* and *hlcust* in the R environment. Correlation analyses were done on the raw data after data conversion to binary outcomes (1 = feature presence, 0 = feature absence). The correlation was estimated on a scale from +1 to −1. The significance of the correlation was assessed at a significance level of 0.05. The variables that have similar occurrences in all isolates were excluded from this analysis. The correlation analyses and visualization were done using R packages *corrplot*, *heatmaply*, *hmisc*, and *ggpubr* [44,45,46]. Fisher’s exact two-tailed test [47] was used to study the infection rates of *S. uberis* among farms of varying hygiene interventions and their antimicrobial resistance; *p*- values ˂ 0.05 were statistically significant.

## 3. Results

### 3.1. Infection Rate and Characterization of S. uberis in Clinically Mastitic Dairy Cows

The overall infection rate of *S. uberis* was 20.59% (69/335), which significantly (*p* < 0.05) differed between farms, being 8.8% (11/125) in animals living in farms with adequately applied hygiene measures and 27.61% (58/210) in animals living on low hygiene, hand machine farms and smallholders. On Edward’s media, *S. uberis* isolates appeared as colorless dewdrop-like, pinpoint rounded colonies. Phenotypic characteristics of the isolates denoted Gram-positive cocci, arranged mainly in chains and, sometimes, in diplococci. They showed β or γ hemolytic colonies on blood agar media. CAMP-like hemolytic activities were determined, together with beta-toxin-producing *Staphylococcus aureus*, on sheep blood agar in 60 out of 69 (86.9%) *S. uberis* isolates. Biochemically, *S. uberis* isolates were catalase-test-negative, whereas all isolates were positive for sodium hippurate and bile-esculin hydrolyses tests. The isolates fail to grow on MacConkey’s agar, media containing 6.5% NaCl, or at 45 °C, which is characteristic for *S. uberis*.

### 3.2. Antimicrobial Resistance Patterns of S. uberis Isolates

**Figure 1:**
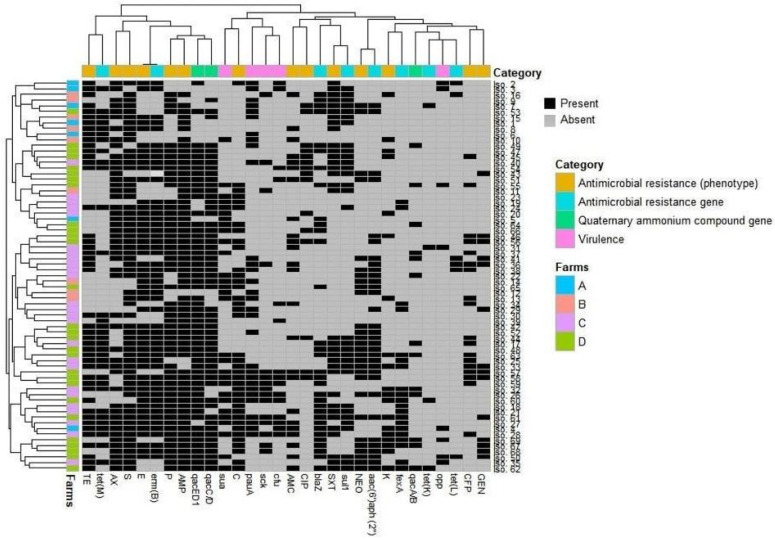
Overall occurrence and clustering of *S. uberis* isolates (*n* = 69) in the investigated farms, their virulence attributes, and antimicrobial and biocide resistance patterns. The heatmap shows the occurrence of features in all isolates. The dendrogram indicates the hierarchical clustering of features and isolates. Different farms and feature categories are color-coded, as shown in the label. GEN, gentamycin; CFP, cefoperazone; K, kanamycin; NEO, neomycin; SXT, trimethoprim-sulfamethoxazole; CIP, ciprofloxacin; AMC, amoxicillin–clavulanic acid; C, chloramphenicol; AMP, ampicillin; P, penicillin; E, erythromycin; S, streptomycin; AX, amoxicillin; TE, tetracycline.

The antimicrobial resistance of *S. uberis* isolates (*n* = 69) was validated against 21 antimicrobials of 12 chemotherapeutic classes. As shown in Table 1 and Figure 1, *S. uberis* exhibited 100% resistance to cloxacillin, ceftriaxone, cephalexin, clindamycin, and novobiocin. Moreover, high levels of resistance were reported for ampicillin (89.85%), streptomycin (86.96%), penicillin (79.71%), and erythromycin (73.91%). On the other hand, kanamycin (30.43%), cefoperazone (26.04%), ciprofloxacin (21.74 %), and gentamycin (20.28%) showed the lowest resistance levels, and none of the isolates exhibited imipenem resistance. Of note, all *S. uberis* isolates were MDR, with MAR indices ranged from 0.38–0.81, whereas the MAR indices for tested antimicrobials were up to 0.048. Statistical analysis revealed a significant variation in the resistance levels of *S. uberis* isolates to various antimicrobial agents (*p* ˂ 0.05).

### 3.3. Molecular Characteristics and Virulence Gene Profiling of S. uberis

Conventional identification of *S. uberis* isolates (*n* = 69) was confirmed by PCR-based amplification of the genus-specific *tuf* gene (DNA fragment ~196 bp). Further, the 16S rRNA gene identified *S. uberis* at the species level (DNA product ~854 bp). *S. uberis* isolates were tested by PCR for the existence of five major genes potentially involved in virulence (Table 2). The most frequent gene was *sua* (42.02%), followed by *pauA* (40.57%), *cfu* (21.73%), and *skc* (20.28%). In contrast, the *opp* gene was detected with a low percentage (11.59%). The frequency of putative virulence gene patterns among *S. uberis* isolates is summarized in Table 3. Most of the examined isolates (58/69; 84.06%) harbored at least one virulence gene. Moreover, 11 of 69 isolates (15.94%) possessed simultaneously 3 to 4 virulence-associated genes, and 7 (10.14%) *S. uberis* isolates carried 2 different virulence-related genes. The most frequent virulence gene pattern was *sua* + *pauA* + *skc* + *cfu*, which was observed in 8 of 69 isolates (11.59%) from 2 different herds (C and D) of low hygiene measures.

### 3.4. Detection of Antimicrobial Resistance Genes in S. uberis Isolates

The detection of antimicrobial resistance genes confirmed the phenotypic resistance patterns of the respective *S. uberis* isolates (Table 2). As presented in Figure 1, the erythromycin resistance gene *erm*(B) was the most prevalent among the analyzed isolates (75.36%). However, *erm*(C) and *erm*(A) genes were not amplified in either erythromycin-resistant or erythromycin-susceptible *S. uberis* isolates. The most frequent tetracycline resistance gene was *tet*(M) (46.38%), whereas *tet*(L) and *tet*(K) genes were recorded in lower frequencies (8.7 and 5.8%, respectively), and the *tet*(O) gene was not detected in any of the tested *S. uberis* isolates. The *blaZ* and *aac(6′)aph(2″)* genes, conferring resistance to penicillins and aminoglycosides, respectively, were similarly found in 32 out of 69 examined isolates (46.38% each). Furthermore, the *fexA* gene, conferring resistance to chloramphenicol, was detected in 20 *S. uberis* isolates (28.99%). The sulfonamide resistance gene, *sul1*, was found in 31 (44.93%) *S. uberis* isolates, but the trimethoprim *dfrA* gene was not detected in any analyzed isolate.

### 3.5. Biocide Resistance Genes and Biocide–Antimicrobial Cross-Resistance

**Figure 2:**
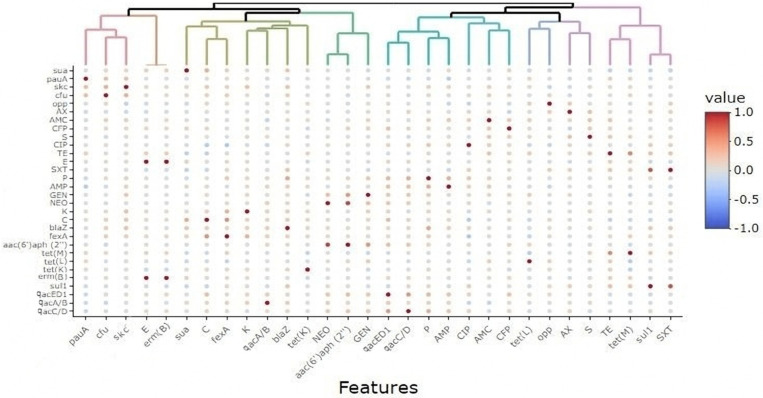
Correlations among various features in *S. uberis* isolates (*n* = 69) from various farms. The color scale represents the correlation coefficient (*R*) on a scale from +1 to −1 (+1 is the highest positive correlation, and −1 is the highest negative correlation). AX, amoxicillin; AMC, amoxicillin–clavulanic acid; CFP, cefoperazone; S, streptomycin; CIP, ciprofloxacin; TE, tetracycline; E, erythromycin; SXT, trimethoprim-sulfamethoxazole; P, penicillin; AMP, ampicillin; GEN, gentamycin; NEO, neomycin; K, kanamycin; C, chloramphenicol.

Biocide resistance profiling showed that 55 out of 69 *S. uberis* isolates (79.71%) carried *qac* genes; among them, 55 (79.71%), 54 (78.26%), and 13 (18.84%) exhibited resistance to *qacED1*, *qacC/D*, and *qacA/B*, respectively. Biocide resistance gene combinations were detected among the isolates; 3 gene combinations were found in 11 (15.94%) isolates, and 2 combinations, either *qacED1* + *qacC/D* (38/69, 55.07%) or *qacED1* + *qacA/B* (2/69, 2.9%), were also reported (Table 2). The selective pressure employed by exposure to biocides may be associated with increasing antimicrobial resistance. As shown in Figure 2 and Table S2, significant (*p* ˂ 0.05) positive correlations (*r* = 0.01–0.43) between QAC tolerance and resistance to various antimicrobials indicate the pervasive occurrence of multi-drug efflux pumps. However, non-significant (*p* ˃ 0.05) negative correlations were observed between the existence of *qac* genes and resistance to certain antimicrobials such as amoxicillin–clavulanate (*r* = −0.01, −0.1 and −0.05), tetracycline (*r* = −0.06, −0.03 and −0.01), and trimethoprim-sulfamethoxazole (*r* = −0.02, −0.01 and −0.05) for *qacED1*, *qacC/D*, and *qacA/B* genes, respectively.

### 3.6. Typing of Virulent S. uberis Isolates Using RFLP–PCR

Phenotypically, *S. uberis* type I and *S. parauberis* (*S. uberis* type II) isolates had similar cultural, morphological, and biochemical characteristics and could not be differentiated by conventional methods. Therefore, RFLP–PCR analysis of the 16S rRNA gene was used to characterize them, and the results indicated that all isolates (*n* = 69) were indeed *S. uberis* (*S. uberis* type I) by their unique RFLP pattern (Figure S1).

### 3.7. Association between the Existence of Virulence Traits, Antimicrobial and Biocide Resistance, and Hygienic Interventions for Dairy Cows

**Figure 3:**
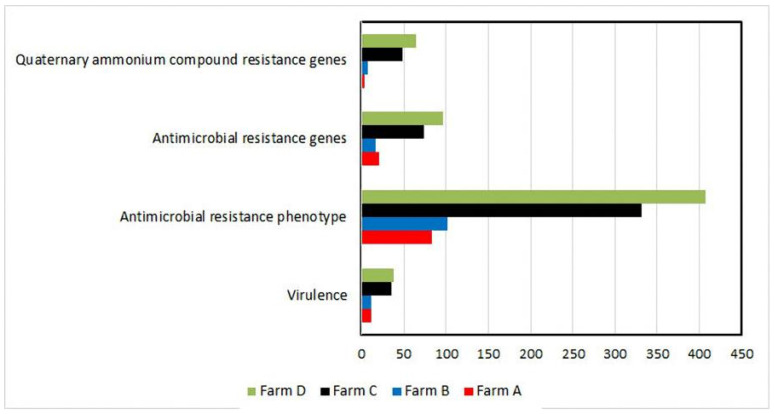
Differences among farms are shown in the term of studied features. Each horizontal bar represents the overall number of isolates (*x*-axis) showing a certain feature (including redundancy). Farms are shown in different colors. Farm D possessed the highest number of isolates harboring the studied features compared to other farms.

As shown in Table 2, the occurrence of virulence-associated and antimicrobial- and QAC resistance genes was distributed among *S. uberis* isolates (*n* = 69) over all the investigated dairy farms. However, the simultaneous existence of four virulence genes (*sua + pauA* + *skc + cfu*, 11.59%), more than four antimicrobial resistance genes (17.39%), and the three tested *qac* genes (*qacA/B + qacED1 + qacC/D*, 15.94%) was reported only in C and D dairy herds. However, nine (13.04%) *S. uberis* isolates recovered from the C and D dairy herds did not carry any QAC resistance genes. Overall, as presented in Figure 3, the four studied features (virulence, resistance phenotype/genes, and *qac* genes) were prominent in the dairy herds with moderate and low hygiene measures (C and D, respectively).

**Figure 4:**
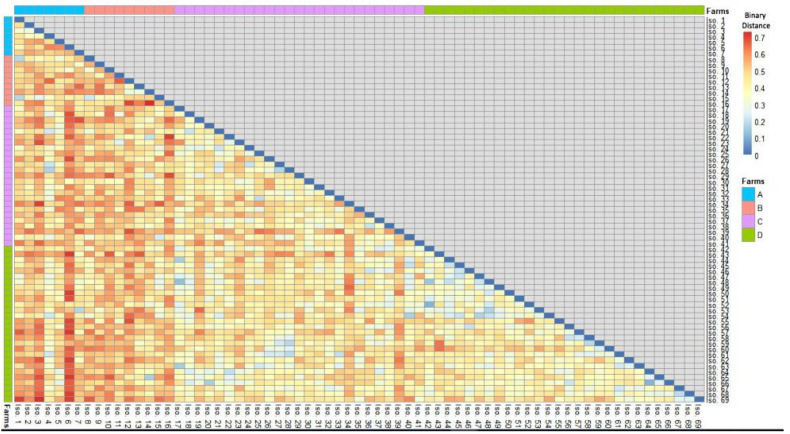
A heatmap showed the binary distances among *S. uberis* isolates based on the presence or absence of the four studied features (Scheme 0.7).

**Figure 5:**
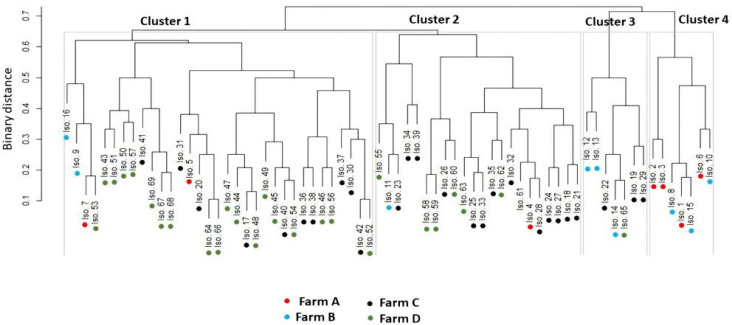
Hierarchical clustering dendrogram showing the relatedness (closeness) of various isolates (shown as numbers) from different farms (shown as colored dots) based on all the feature categories. All isolates were classified into four clusters.

Figure 4 and Table S3 demonstrate that *S. uberis* isolates (*n*= 69) had a low-to-moderate diversity (Euclidean distance = 0.11–0.73) among the investigated dairy herds. The dendrogram analysis (Figure 5) classified the isolates into four clusters (1, 2, 3, and 4). A close relatedness was noticed among certain *S. uberis* isolates from different dairy herds. Most *S. uberis* isolated from C and D dairy herds were closely related and gathered in clusters 1 and 2. In addition, *S. uberis* of A and B dairy herds were clustered closely in cluster 4. Few isolates of the four dairy herds clustered together in cluster 3.

## 4. Discussion

Mastitis remains a critical problem for dairy animals, causing drastic losses during lactation seasons. Such losses are attributed mainly to decreased milk yield, lower milk quality, and higher treatment and control costs [48]. *S. uberis* is a well-known pathogen that causes bovine intramammary infections worldwide. Nonetheless, there are scant epidemiological data on *S. uberis* isolated from lactating cows in Egypt, especially in the smallholder production system, despite the fact that this extensive system type is the most common traditional livestock farming system among Egyptian farmers [49,50].

In the current study, the overall infection rate of *S. uberis* in dairy cows of different parity, showing gross signs of clinical mastitis associated with or without systemic reactions, was 20.59%, which is nearly similar to a previously published work (23.5%) [51]. Comparatively lower infection rates (6.3%, 9.3%, and 11.8%, respectively) of *S. uberis* isolated from mastitic cattle were previously reported by several studies [52,53,54]. Higher rates of *S. uberis* infection (55.38 and 33%, respectively) were reported in previous studies [55,56]. Our findings may represent a potential hygiene deficiency that has a significant role in the occurrence of environmental *S. uberis* mastitis [57].

Animals with adequate hygiene during milking (A and B dairy herds) had a lower prevalence of infection (11/125; 8.8%) than those with poor hygiene (C and D) during the milking process (58/210; 27.61%). The predominance of the microorganisms varies according to the handling practices of the animals and the hygiene conditions during milking [58]. The lower infection rate may be attributed to good management practices such as the milkers’ hygiene, sanitization of the milking machine, healthy udder environment, dry period treatment, and the control of other predisposing diseases. Meanwhile, the higher infection rate (herds C, D) may be ascribed to a group of shared breeding factors where the dairy cattle live, including bad habitats, unbalanced food, terrible drafts, and the lack of pre-milking procedures. These conditions play a role in rendering the udder more susceptible to intramammary infections [59]. Furthermore, variations in the microenvironments and management practices between the different hygiene features applied to farms can influence the existence of the disease.

Herein, 21 antimicrobials of 12 antimicrobial classes were chosen to be tested, considering their availability for the intramammary treatment of clinical mastitis. In addition, we monitored penicillin, phenicol, aminoglycoside, tetracycline, sulfonamide, and trimethoprim resistance phenotypes/genotypes among the bovine mastitis *S. uberis* isolates.

Penicillin is widely used in the treatment of clinical bovine mastitis. The proportions of ampicillin-, penicillin-, and amoxicillin-resistant isolates in this study were high (89.85%, 79.71%, and 69.57%, respectively). Our finding strongly supported the previous results of Haenni et al. [60], who described a shift toward penicillin resistance among a subpopulation of *S. uberis* isolates. Additionally, they identified the presence of resistance-associated mutations among isolates considered intermediately susceptible to penicillin. Here, the *blaZ* gene conferring resistance to penicillin was found in 46.38% of the examined isolates, indicating an alarming level of potential resistance in bovine mastitis. This finding conflicts with the claim that environmental streptococci are still susceptible to β-lactam-active substances [61]. Previous studies [62,63] have documented that penicillin is effective against streptococci isolates with percentages of 92% and 96%, respectively. Moreover, Minst and coauthors [64] noticed the absence of penicillin and ampicillin resistance, suggesting that β-lactam antibiotics should remain the drug of choice for treating streptococcal mastitis. In this study, a low level of gentamicin resistance (20.28%) against *S. uberis* isolates was observed. On the contrary, an earlier study [65] reported that up to 93% of streptococci were resistant to gentamicin. Additionally, Rato et al. [66] stated that most *S. uberis* isolates (80%) were resistant to gentamycin. Nevertheless, our results are comparable to a lower rate of gentamicin-resistant *S. uberis* in a previous German study [67]. On the other hand, the *S. uberis* resistance rate to streptomycin was 96%, precluding its use in the treatment of bovine mastitis, which is consistent with a previous study [66]. The aminoglycoside resistance gene, *aac(6′)aph(2″)*, was detected here within a reasonable rate (46.38%), which provides evidence suggesting that it confers resistance to a broad spectrum of aminoglycosides in Gram-positive bacteria, including streptococci [68], whereas the *aac(6′)-Ib* gene confers resistance to tobramycin, kanamycin, and amikacin in Gram-negative bacteria [69].

Our results showed that *S. uberis* is highly resistant to erythromycin (73.91%), which is higher than previous reports from France (21%) [70], Argentina (27.6%) [71], and northwestern China (31.2%) [15]. This explains that the *erm*(B) gene is the most prevalent among the analyzed isolates (75.36%). Furthermore, 65.22% of *S. uberis* isolates displayed resistance against tetracycline, mainly due to the inclusion of the *tet*(M) gene (46.38%) in most resistant isolates, which is nearly similar to a previous report (60%) [66]. However, the levels in our findings were lower than that previously described in a previous research (81.3%) [15]. Another study performed on *S. uberis* isolates from dairy cattle with clinical mastitis found results lower than ours for tetracycline resistance (18.1%) [72]. High tetracycline resistance levels may be attributed to their widespread use in treating numerous cattle infections for several years, proposing that tetracyclines, quinolones, and aminoglycosides should be avoided for the treatment of streptococcal mastitis. Differences in the susceptibility patterns among various studies could be due to different antimicrobial use in farms or countries, which could be a consequence of antimicrobial overuse for treating clinical mastitis or for growth promotion purposes in dairy herds [14], thus resulting in the inclusion of drug-resistant bacteria even in raw milk [73].

In the present investigation, conventional PCR allowed the amplification of virulence-associated genes of *S. uberis*, namely, *sua*, *skc*, *cfu*, *pauA*, and *opp*, each represented by a single band to their respective base pairs in the corresponding region of the DNA marker. The detection of genes encoding virulence factors could explain a possible association in the pathogenesis of mammary infections. The CAMP gene (*cfu*) is recorded here with a percentage of 21.73%; nearly similar results have been reported (25%) [74]. However, previous studies have reported high frequencies of the *cfu* gene in *S. uberis* isolates: 76.9% [11], 55.5% [53], and 46.1% [52]. On the contrary, a lower *cfu* percentage (3.8%) was reported in a previous research article [7]. The results suggest that this gene might not be the only gene related to the expression of the CAMP reaction. The *opp* gene was found in 11.59% of the examined *S. uberis* isolates. Previous studies have described a higher percentage of *opp* in *S. uberis* isolates: 64.1% [11] and 22.2% [53]. In contrast, an earlier study reported that the *opp* gene could not be amplified from all the strains, suggesting this gene may not be the only one responsible for the growth of *S. uberis* in milk [75]. The *pauA* gene was found in 40.57% of the examined *S. uberis* isolates. On the contrary, Ward et al. [76] reported that expression of *pauA* is not essential for infection of the mammary gland, as none of the examined isolates harbored the *pauA* gene from mastitic cows in an experimental study. In the same way, previous reports [10,11] found the *pauA* gene in *S. uberis* isolates with a higher percentage (94.9% and 61.5%, respectively). The streptokinase gene (*skc*) was detected at a percentage of 20.28%. A higher result was recorded by Shome and coauthors [52], who reported the *skc* gene in *S. uberis* strains at an incidence of 100%. The *sua* gene was recorded in our research at 42.02%. Nearly similar results were obtained previously (38.5%) [52]. In contrast, higher rates (97.8% and 83.3%, respectively) have been previously recorded [10,11].

Quaternary ammonium compounds (QACs) are amongst the most frequently used disinfectants. They are known to hinder the activity of a broad spectrum of microorganisms. They can disrupt the microbial cell wall, resulting in the leaking of the cytoplasm out of the cells [77]. Regrettably, the prevailing usage of QAC-based antiseptics in animal husbandry may result in bacterial resistance. In this study, QAC resistance genes were examined in *S. uberis* isolates. The *qacED1* (79.71%) and *qacC/D* (78.26%) genes were found more frequently than *qacA/B* (18.84%). A paucity of data is currently available regarding the extent of QAC resistance genes in environmental streptococcal mastitis in Egypt. In a previous study in Egypt, all examined *S. uberis* isolates from bovine mastitis showed 100% phenotypic resistance to QACs (TH4; concentration = 0.25%) [78]. However, there are no Egyptian reports on QAC resistance in *S. uberis* isolates at the genetic level. The selective pressure employed by exposure to biocides has been concomitant with increasing resistance to antimicrobial agents. It has been documented that biocides and antimicrobial agents may share joint target sites and be situated together in mobile elements, resulting in co-resistance [79]. In addition, chromosomal efflux pumps may be involved in antimicrobial and biocide resistance due to their non-specific mechanism [80]. Inconsistent with a previous investigation on biocide resistance in *Staphylococcus aureus* [81], we report a weak or moderate genetic correlation between the existence of QAC and antimicrobial resistance genes.

## 5. Conclusions

The present investigation is the first to look into the virulence attributes and genotypic resistance to antimicrobials and biocides in *S. uberis* isolates from bovine clinical mastitis in Egypt. It adds to our knowledge of the high diversity of *S. uberis* and its occurrence in relation to prospective hygienic concerns. The sustained presence of *pauA* and *sua* genes throughout the investigated farms contributes to a better understanding of the pathogenicity of the bacterium, which provides the need to use such virulence factors as potential constituents of a vaccine against *S. uberis*. The co-existence of MDR and biocide resistance indicates the importance of *S. uberis* surveillance and the prudent use of antimicrobials and antiseptics in veterinary clinical medicine to avoid the dissemination of resistance.

## Figures and Tables

**Table 1:** Antimicrobial resistance of *S. uberis* isolated from lactating cows with clinical mastitis.

Antimicrobial Class	AMA	No. of Resistant Isolates (%)	MAR Index	Fisher Exact *p*-Value *
Beta-lactams	CX	69 (100.00)	0.048	NE
	AMP	62 (89.85)	0.043	0.007
	AX	48 (69.57)	0.033	<0.001
	P	55 (79.71)	0.038	<0.001
Beta-lactamase inhibitor	AMC	24 (34.78)	0.017	<0.001
Cephalosporins	CRO	69 (100.00)	0.048	NE
	CFP	18 (26.09)	0.012	<0.001
	CL	69 (100.00)	0.048	NE
	FEP	69 (100.00)	0.048	NE
Non-beta Lactams (Carbapenems)	IPM	0 (00.00)	0.00	<0.001
Lincomycins	DA	69 (100.00)	0.048	NE
Fluoroquinlones	CIP	15 (21.74)	0.010	<0.001
Tetracyclines	TE	45 (65.22)	0.031	<0.001
Macrolides	E	51(73.91)	0.035	0.001
Aminoglycosides	S	60 (86.96)	0.041	0.002
	GEN	14 (20.28)	0.009	<0.001
	NEO	28 (40.57)	0.019	<0.001
	K	21 (30.43)	0.014	<0.001
Phenicols	C	38 (55.07)	0.026	<0.001
Aminocoumarins	NV	69 (100.00)	0.048	NE
Sulfonamides	SXT	33 (47.83)	0.023	<0.001

MAR, multiple antibiotic resistance index; AX, amoxicillin; AMC, amoxicillin–clavulanic acid; CX, cloxacillin; CRO, ceftriaxone; CFP, cefoperazone; CL, cephalexin; FEP, cefepime; IPM, imipenem; S, streptomycin; DA, clindamycin; CIP, ciprofloxacin; TE, tetracycline; E, erythromycin; NEO, neomycin; P, penicillin; NV, novobiocin; AMP, ampicillin; GEN, gentamycin; C, chloramphenicol; K, kanamycin; SXT, trimethoprim–sulfamethoxazole; NE, not estimated. * *p*-value < 0.01 was considered highly statistically significant.

**Table 2:** Virulence traits and antimicrobial and biocide resistance profiles of *S. uberis* (*n* = 69) isolated from dairy cows of different hygiene interventions.

Isolate No.	Herd	Virulence Pattern	Antimicrobial Resistance Profile		Biocide Resistance Genes
			Phenotype	Resistance Genes	
1	A	ND	CX, CRO, CL, FEP, S, DA, TE, E, SXT, AMP, NV	*tet*(M), *erm*(B), *sul1*	ND
2		*pauA*, *cfu*, *opp*	AX, CX, CRO, CL, FEP, S, DA, TE, E, SXT, AMP, NV, C	*tet*(L), *erm*(B)	*qacED1*
3		*pauA*, *cfu*, *opp*	AX, CX, CRO, CL, FEP, DA, TE, E, SXT, NV	*tet*(M), *erm*(B), *sul1*	ND
4		*pauA*, *cfu*, *opp*	AX, AMC, CX, CRO, CL, FEP, S, DA, TE, E, SXT, P, AMP, NV, K, C	*blaZ*, *fexA*, *tet*(M), *sul1*	*qacED1*, *qacC/D*
5		*sua*	AX, AMC, CX, CRO, CL, FEP, S, DA, E, P, AMP, NV	*blaZ*, *erm*(B)	ND
6		*pauA*	CX, CRO, CL, FEP, S, DA, TE, NV	*tet*(M)	ND
7		*pauA*	AX, CX, CRO, CL, FEP, S, DA, CIP, TE, SXT, P, AMP, NV, NEO	*blaZ*, *aac(6′)aph(2″)*, *tet*(K), *sul1*	ND
8	B	ND	AX, AMC, CX, CRO, CL, FEP, S, DA, TE, E, SXT, AMP, NV	*tet*(M), *erm*(B)	ND
9		*pauA*	AX, CX, CRO, CL, FEP, S, DA, SXT, P, NV	*blaZ*, *sul1*	*qacC/D*
10		*sua*, *pauA*	AX, AMC, CX, CRO, CL, FEP, S, DA, TE, AMP, NV, K	*tet*(M)	ND
11		*skc*	AX, CX, CRO, CL, FEP, S, DA, SXT, AMP, NV, C	*sul1*	*qacED1*, *qacC/D*
12		*sua*, *pauA*	CX, CRO, CL, FEP, S, DA, E, NV, NEO	*aac(6′)aph(2″)*, *erm*(B)	ND
13		*sua*, *pauA*	CX, CRO, CFP, CL, FEP, S, DA, E, AMP, NV, K, C	*erm*(B)	*qacED1*
14		*sua*, *pauA*	CX, CRO, CL, FEP, DA, E, AMP, NV, NEO, C	*aac(6′)aph(2″)*, *erm*(B)	*qacED1*, *qacC/D*
15		*pauA*	AX, CX, CRO, CL, FEP, S, DA, TE, E, SXT, AMP, NV	*tet*(M), *erm*(B), *sul1*	*qacC/D*
16		*skc*	AMC, CX, CRO, CL, FEP, S, DA, TE, SXT, P, AMP, NV, K	*blaZ*, *tet*(L), *sul1*	ND
17	C	ND	CX, CRO, CFP, CL, FEP, S, DA, TE, E, SXT, P, AMP, NV, NEO	*blaZ*, *aac(6′)aph(2″)*, *tet*(M), *erm*(B), *sul1*	*qacA/B*, *qacED1*, *qacC/D*
18		ND	AX, CX, CRO, CL, FEP, S, DA, E, SXT, P, AMP, NV, C	*blaZ*, *fexA*, *erm*(B), *sul1*	*qacED1*, *qacC/D*
19		*sua*	CX, CRO, CL, FEP, DA, E, P, AMP, NV, C	*fexA*, *erm*(B)	*qacC/D*
20		*sua*	AX, CX, CRO, CL, FEP, S, DA, CIP, E, P, AMP, NV, K	*erm*(B)	*qacED1*, *qacC/D*
21		*sua*	AX, AMC, CX, CRO, CL, FEP, S, DA, TE, E, SXT, P, AMP, NV, C	*blaZ*, *fexA*, *tet*(M), *erm*(B), *sul1*	*qacED1*
22		*sua*	AX, CX, CRO, CL, FEP, S, DA, E, AMP, NV, NEO, C	*aac(6′)aph(2″)*, *erm*(B)	*qacA/B*, *qacED1*, *qacC/D*
23		*sua*	AX, CX, CRO, CL, FEP, S, DA, AMP, NV, C	ND	*qacED1*, *qacC/D*
24		*sua*	AX, CX, CRO, CL, FEP, S, DA, TE, E, P, AMP, NV, C	*fexA*, *tet*(M), *erm*(B), *sul1*	*qacC/D*
25		*sua*	AX, CX, CRO, CFP, CL, FEP, S, DA, TE, E, SXT, NV, NEO, C	*fexA*, *aac(6′)aph(2″)*, *tet*(M), *erm*(B), *sul1*	*qacED1*, *qacC/D*
26		*sua*, *pauA*, *skc*, *cfu*	CX, CRO, CL, FEP, S, DA, E, SXT, P, AMP, NV, NEO, K, C	*blaZ*, *fexA*, *aac(6′)aph(2″)*, *erm*(B)	*qacA/B*, *qacED1*
27		*sua*, *pauA*, *skc*, *cfu*	AX, AMC, CX, CRO, CL, FEP, S, DA, TE, E, P, AMP, NV, C	*blaZ*, *fexA*, *tet*(M), *erm*(B), *sul1*	*qacC/D*
28		*sua*, *pauA*, *skc*, *cfu*	AX, CX, CRO, CFP, CL, FEP, S, DA, TE, E, SXT, P, AMP, NV, K, C	*blaZ*, *fexA*, *tet*(M), *erm*(B)	*qacED1*, *qacC/D*
29		*pauA*	CX, CRO, CL, FEP, DA, E, P, AMP, NV, GEN, C	*fexA*, *aac(6′)aph(2″)*, *erm*(B)	*qacED1*, *qacC/D*
30		*pauA*	AX, CX, CRO, CL, FEP, S, DA, TE, AMP, NV, K, C	*tet*(M)	*qacED1*, *qacC/D*
31		*opp*	AX, AMC, CX, CRO, CL, FEP, S, DA, TE, E, P, AMP, NV, C	*tet*(K), *erm*(B)	*qacED1*, *qacC/D*
32		*pauA*, *skc*	AX, CX, CRO, CL, FEP, S, DA, TE, E, P, AMP, NV, K, C	*fexA*, *tet*(M), *erm*(B)	*qacA/B*, *qacED1*
33		*pauA*	AX, CX, CRO, CFP, CL, FEP, S, DA, TE, E, SXT, P, NV, GEN, NEO, K, C	*fexA*, *aac(6′)aph(2″)*, *tet*(M), *erm*(B), *sul1*	*qacED1*, *qacC/D*
34		*cfu*	AMC, CX, CRO, CFP, CL, FEP, S, DA, P, AMP, NV, C	*fexA*	*qacED1*
35		*opp*	AX, CX, CRO, CL, FEP, S, DA, TE, SXT, P, AMP, NV, NEO, C	*fexA*, *aac(6′)aph(2″)*, *tet*(M), *sul1*	*qacED1*, *qacC/D*
36		*pauA*, *skc*	AMC, CX, CRO, CFP, CL, FEP, S, DA, TE, E, P, AMP, NV, GEN	*aac(6′)aph(2″)*, *tet*(L), *erm*(B)	*qacED1*, *qacC/D*
37		*sua*	AX, CX, CRO, CFP, CL, FEP, S, DA, TE, E, P, AMP, NV	*tet*(M), *erm*(B)	*qacA/B*, *qacED1*, *qacC/D*
38		*pauA*	AX, AMC, CX, CRO, CFP, CL, FEP, S, DA, TE, E, P, AMP, NV, NEO, C	*aac(6′)aph(2″)*, *tet*(L), *erm*(B)	*qacED1*, *qacC/D*
39		*cfu*	CX, CRO, CL, FEP, DA, P, AMP, NV	*tet*(M)	*qacED1*, *qacC/D*
40		*pauA*, *skc*	AX, AMC, CX, CRO, CL, FEP, S, DA, CIP, TE, E, SXT, P, AMP, NV	*tet*(M), *erm*(B), *sul1*	*qacED1*, *qacC/D*
41		*pauA*	AX, CX, CRO, CL, FEP, S, DA, TE, P, AMP, NV, GEN, NEO	*aac(6′)aph(2″)*, *tet*(L)	*qacA/B*, *qacED1*, *qacC/D*
42	D	ND	AX, CX, CRO, CL, FEP, S, DA, TE, E, P, AMP, NV, NEO	*aac(6′)aph(2″)*, *tet*(M), *erm*(B)	*qacED1*, *qacC/D*
43		ND	AX, CX, CRO, CL, FEP, DA, CIP, SXT, P, AMP, NV, GEN, NEO	*aac(6′)aph(2″)*, *sul1*	*qacED1*, *qacC/D*
44		ND	AMC, CX, CRO, CFP, CL, FEP, S, DA, CIP, TE, E, SXT, P, AMP, NV, NEO	*aac(6′)aph(2″)*, *tet*(M), *erm*(B), *sul1*	*qacED1*, *qacC/D*
45		ND	AX, AMC, CX, CRO, CFP, CL, FEP, S, DA, CIP, TE, E, SXT, P, AMP, NV, K	*erm*(B), s*ul1*	*qacED1*, *qacC/D*
46		ND	AX, AMC, CX, CRO, CFP, CL, FEP, S, DA, TE, E, P, AMP, NV, GEN, K	*aac(6′)aph(2″)*, *erm*(B)	*qacED1*, *qacC/D*
47		ND	CX, CRO, CL, FEP, S, DA, CIP, TE, E, SXT, P, AMP, NV, K	*blaZ*, *tet*(M), *erm*(B), *sul1*	*qacED1*, *qacC/D*
48		ND	AX, CX, CRO, CL, FEP, S, DA, TE, E, SXT, P, AMP, NV, NEO	*blaZ*, *aac(6′)aph(2″)*, *tet*(M), *erm*(B), *sul1*	*qacED1*, *qacC/D*
49		*pauA*	AX, CX, CRO, CL, FEP, DA, CIP, TE, E, SXT, P, AMP, NV	*blaZ*, *erm*(B), *sul1*	*qacA/B*, *qacED1*, *qacC/D*
50		*sua*, *opp*	AX, AMC, CX, CRO, CL, FEP, S, DA, TE, E, SXT, P, AMP, NV, GEN, NEO	*blaZ*, *aac(6′)aph(2″)*, *tet*(L), *erm*(B), *sul1*	*qacED1*, *qacC/D*
51		*cfu*	AX, AMC, CX, CRO, CL, FEP, S, DA, CIP, E, P, AMP, NV, NEO	*aac(6′)aph(2″)*, *erm*(B)	*qacED1*, *qacC/D*
52		*pauA*	CX, CRO, CL, FEP, S, DA, TE, E, P, AMP, NV, NEO	*aac(6′)aph(2″)*, *tet*(M), *erm*(B)	*qacED1*, *qacC/D*
53		*pauA*	AX, CX, CRO, CL, FEP, S, DA, CIP, TE, SXT, P, AMP, NV, NEO	*blaZ*, *aac(6′)aph(2″)*, *tet*(M), *sul1*	*qacED1*, *qacC/D*
54		*cfu*	AX, AMC, CX, CRO, CL, FEP, S, DA, CIP, TE, E, SXT, P, AMP, NV	*blaZ*, *tet*(M), *erm*(B), *sul1*	*qacED1*, *qacC/D*
55		*sua*, *opp*	AX, CX, CRO, CFP, CL, FEP, S, DA, SXT, P, AMP, NV, C	*blaZ*, *sul1*	*qacA/B*, *qacED1*, *qacC/D*
56		*sua*	AX, AMC, CX, CRO, CFP, CL, FEP, S, DA, CIP, TE, E, P, AMP, NV, GEN, C	*blaZ*, *aac(6′)aph(2″)*, *erm*(B)	*qacED1*, *qacC/D*
57		*sua*, *pauA*, *skc*, *cfu*	AX, AMC, CX, CRO, CFP, CL, FEP, S, DA, CIP, SXT, P, AMP, NV, GEN, NEO, C	*blaZ*, *aac(6′)aph(2″)*, *sul1*	*qacED1*, *qacC/D*
58		*sua*, *pauA*, *skc*, *cfu*	AMC, CX, CRO, CFP, CL, FEP, S, DA, CIP, TE, E, P, AMP, NV, GEN, NEO, C	*blaZ*, *aac(6′)aph(2″)*, *tet*(M), *erm*(B)	*qacED1*, *qacC/D*
59		*sua*, *pauA*, *skc*, *cfu*	CX, CRO, CFP, CL, FEP, S, DA, TE, E, P, AMP, NV, C	*blaZ*, *tet*(M), *erm*(B)	*qacED1*, *qacC/D*
60		*sua*, *pauA*, *skc*, *cfu*	CX, CRO, CL, FEP, DA, TE, E, P, AMP, NV, K, C	*blaZ*, *fexA*, *tet*(K), *erm*(B)	*qacED1*, *qacC/D*
61		*sua*, *pauA*, *skc*, *cfu*	AX, CX, CRO, CL, FEP, S, DA, TE, E, SXT, P, AMP, NV, GEN, NEO, K, C	*blaZ*, *fexA*, *aac(6′)aph(2″)*, *tet*(M), *erm*(B), *sul1*	*qacED1*, *qacC/D*
62		*sua*	AX, AMC, CX, CRO, CL, FEP, S, DA, TE, SXT, P, AMP, NV, NEO, K, C	*blaZ*, *fexA*, *aac(6′)aph(2″)*, *tet*(K), *sul1*	*qacA/B*, *qacED1*, *qacC/D*
63		*sua*	CX, CRO, CFP, CL, FEP, S, DA, TE, E, SXT, P, AMP, NV, NEO, K, C	*blaZ*, *fexA*, *aac(6′)aph(2″)*, *tet*(M), *erm*(B), *sul1*	*qacA/B*, *qacED1*, *qacC/D*
64		*sua*	AX, CX, CRO, CL, FEP, S, DA, E, P, AMP, NV, NEO, C	*blaZ*, *erm*(B)	*qacA/B*, *qacED1*, *qacC/D*
65		*sua*	CX, CRO, CL, FEP, DA, E, P, AMP, NV, C	*blaZ*, *aac(6′)aph(2″)*, *erm*(B)	*qacED1*, *qacC/D*
66		*sua*	AX, CX, CRO, CL, FEP, S, DA, CIP, E, P, AMP, NV, C	*blaZ*, *erm*(B)	*qacED1*, *qacC/D*
67		*skc*	AX, AMC, CX, CRO, CL, FEP, S, DA, TE, E, P, AMP, NV, GEN, NEO, K, C	*blaZ*, *fexA*, *aac(6′)aph(2″)*, *tet*(M), *erm*(B)	*qacA/B*, *qacED1*, *qacC/D*
68		*skc*	AX, CX, CRO, CL, FEP, S, DA, E, P, AMP, NV, GEN, NEO, K, C	*blaZ*, *aac(6′)aph(2″)*, *erm*(B)	*qacED1*, *qacC/D*
69		*opp*	AX, CX, CRO, CL, FEP, S, DA, P, AMP, NV, GEN, NEO, K, C	*blaZ*, *fexA*, *aac(6′)aph(2″)*,	*qacA/B*, *qacED1*, *qacC/D*

AX, amoxicillin; AMC, amoxicillin–clavulanic acid; CX, cloxacillin; CRO, ceftriaxone; CFP, cefoperazone; CL, cephalexin; FEP, cefepime; IPM, imipenem; DA, clindamycin; CIP, ciprofloxacin; TE, tetracycline; E, erythromycin; NEO, neomycin; P, penicillin; NV, novobiocin; AMP, ampicillin; S, streptomycin; GEN, gentamycin; C, chloramphenicol; K, kanamycin; SXT, trimethoprim-sulfamethoxazole; ND, not detected.

**Table 3:** Virulence gene profiles of *S. uberis* isolated from lactating cattle experience clinical mastitis.

Molecular Pathotype	Virulence Genes	No. of *S. uberis* Isolates (%)	Farms
I	*sua*, *pauA*, *skc*, *cfu*	8 (11.59)	C, D
II	*pauA*, *cfu*, *opp*	3 (4.35)	A
III	*sua*, *pauA*	4 (5.8)	B
IV	*pauA*, *skc*	3 (4.35)	C
V	*sua*, *opp*	2 (2.9)	D
VI	*sua*	15 (21.74)	A, C, D
VII	*pauA*	10 (14.49)	A, B, C, D
VIII	*skc*	3 (4.35)	B, D
IX	*cfu*	4 (5.8)	C, D
X	*opp*	3 (4.35)	C, D

## Supplementary Materials

The followings are available online at https://www.mdpi.com/article/10.3390/ani11071849/s1, Figure S1: RFLP–PCR analysis of the 16S rRNA gene of representative *S. uberis* isolates showed unique patterns, Table S1: Oligonucleotide primer sequences used for PCR assays, Table S2: Significance and correlations among pairs of the four features under study, Table S3: Calculated binary distances among isolates based on the presence or absence of the four studied features.
