# Supplementary material for: Environmental Streptococcus uberis Associated with Clinical Mastitis in Dairy Cows: Virulence Traits, Antimicrobial and Biocide Resistance, and Epidemiological Typing
Source: Animals (Basel). 2021 Jun 22;11(7):1849. doi: 10.3390/ani11071849 (PMC8300258; doi:10.3390/ani11071849)
Supplement: Supplementary file 1 [file animals-11-01849-s001.zip › Supplementary Materials.pdf]

Supplementary Materials

Table S1. Oligonucleotide primer sequences used for PCR assays.

Table S2 a and b. Significance and correlations among pairs of the of the four features under study.

Table S3. Calculated binary distances among isolates based on the presence or absence of the four-studied features.

Figure S1. RFLP-PCR analysis of 16S rRNA gene of representive *S. uberis* isolates showed unique patterns. Lanes 1-10: *S. uberis* type I isolates, M: molecular weight DNA marker, P: positive control, N: negative control.
