# Supplementary material for: Environmental Streptococcus uberis Associated with Clinical Mastitis in Dairy Cows: Virulence Traits, Antimicrobial and Biocide Resistance, and Epidemiological Typing
Source: Animals (Basel). 2021 Jun 22;11(7):1849. doi: 10.3390/ani11071849 (PMC8300258; doi:10.3390/ani11071849)
Supplement: Supplementary file 1 [file animals-11-01849-s001.zip › Table-S1.pdf]

Table S1. Oligonucleotide primer sequences used for PCR assays

Primer use	Target gene	Nucleotide sequence (5'→3')	Annealing temperature (°C)	Amplicon size (bp)	References
**Streptococci identification**					
Streptococci species	*tuf*	F: GTACAGTTGCTTCAGGACGTATC<br>R:ACGTTCGATTTCATCACGTTG	50	196	[28]
S. uberis	16S rRNA	F:GTACCCTATTTAAAAGGGGCAAAT<br>R:CTCCGATGTACCGAAGTAAAGCTCT	49.5	854	[29]
**Virulence related genes**					
CAMP factor	*cfu*	F:TATCCCGATTTGCAGCCTAC<br>R:CCTGGTCAACTTGTGCAACTG	50	205	[11]
Oligopeptide binding protein	*opp*	F:GGCCTAACCAAAACGAAACA<br>R:GGCTCTGGAATTGCTGAAAG	54	419	[9]
Adhesion molecule	*sua*	F:ACGCAAGGTGCTCAAGAGTT<br>R:TGAACAAGCGATTCGTCAAG	58	776	Accession number DQ232760
Plasminogen activator	*pau*A	F:TGCTACTCAACCATCAAAGGTTGC<br>R:TAGCAGTCTCAGTAGGATGAGTGA	57	439	[30]
Streptokinase activator	*skc*	F:TCCGGATTTTGGGTCCTTAGCCA<br>R:AGTCGACTTTGCGCCTGATGCAC	52	475	[30]
**Antimicrobial resistance genes**					
Penicillins	*blaZ*	F: AAGAGATTTGCCTATGCTTC<br>R: GCTTGACCACTTTTATCAGC	45	517	[31]
Phenicols	*fexA*	F: GTACTTGTAGGTGCAATTACGGCTGA<br>R: CGCATCTGAGTAGGACATAGCGTC	56	1272	[32]
Aminoglycosides	*aac(6')aph (2'')*	F: GAAGTACGCAGAAGAGA<br>R: ACATGGCAAGCTCTAGGA	54	491	[33]
Tetracyclines	*tet*(M)	F: GTGGACAAAGGTACAACGAG<br>R: CGGTAAAGTTCGTCACACAC	50	406	[34]
	tet(O)	F: AACTTAGGCATTCTGGCTCAC<br>R: TCCCACTGTTCCATATCGTCA	56	515	[35]
	tet(L)	F: TCGTTAGCGTGCTGTCATTC<br>R: GTATCCCACCAATGTAGCCG	50	267	[34]
	tet(K)	F: TCGATAGGAACAGCAGTA<br>R: CAGCAGATCCTACTCCTT	44	169	[34]
Macrolides	*erm*(A)	F: GTTCAAGAACAATCAATACAGAG<br>R: GGATCAGGAAAAGGACATTTTAC	48	421	[36]
	erm(B)	F: CATTTAACGACGAAACTGGC<br>R: GGAACATCTGTGGTATGGCG	51	425	[37]
	erm(C)	F: ATCTTTGAAATCGGCTCAGG<br>R: CAAACCCGTATTCCACGATT	47	295	[38]
Sulphonamide	*sul1*	F: CGGCGTGGGCTACCTGAACG<br>R: GCCGATCGCGTGAAGTTCCG	60	433	[39]
Trimethoprim	*dfrA*	F: TGGTAGCTATATCGAAGAATGGAGT<br>R: TATGTTAGAGGCGAAGTCTTGGGTA	60	425	[40]
**Biocide resistance genes**					
Quaternary ammonium compounds	*qacA/B*	F: GCAGAAAGTGCAGAGTTCG<br>R: CCAGTCCAATCATGCCTG	53	361	[41]
	qacC/D	F: GCCATAAGTACTGAAGTTATTGGA<br>R: GACTACGGTTGTTAAGACTAAACCT	53	195	[41]
	qacED1	F:TAAGCCCTACACAAATTGGGAGATAT<br>R:GCCTCCGCAGCGACTTCCACG	58	362	[42]
**PCR-RFLP**					
	16S rRNA	F:GAGAGTTTGATCCTGGCTCAGGA<br>R:CGGGTGTTACAAACTCTCGTGGT	55	1400	[29]

PCR-RFLP, polymerase chain reaction- restriction fragment length polymorphism; bp, base pair
