# Supplementary figures and images for: Environmental Streptococcus uberis Associated with Clinical Mastitis in Dairy Cows: Virulence Traits, Antimicrobial and Biocide Resistance, and Epidemiological Typing
Source: Animals (Basel). 2021 Jun 22;11(7):1849. doi: 10.3390/ani11071849 (PMC8300258; doi:10.3390/ani11071849)

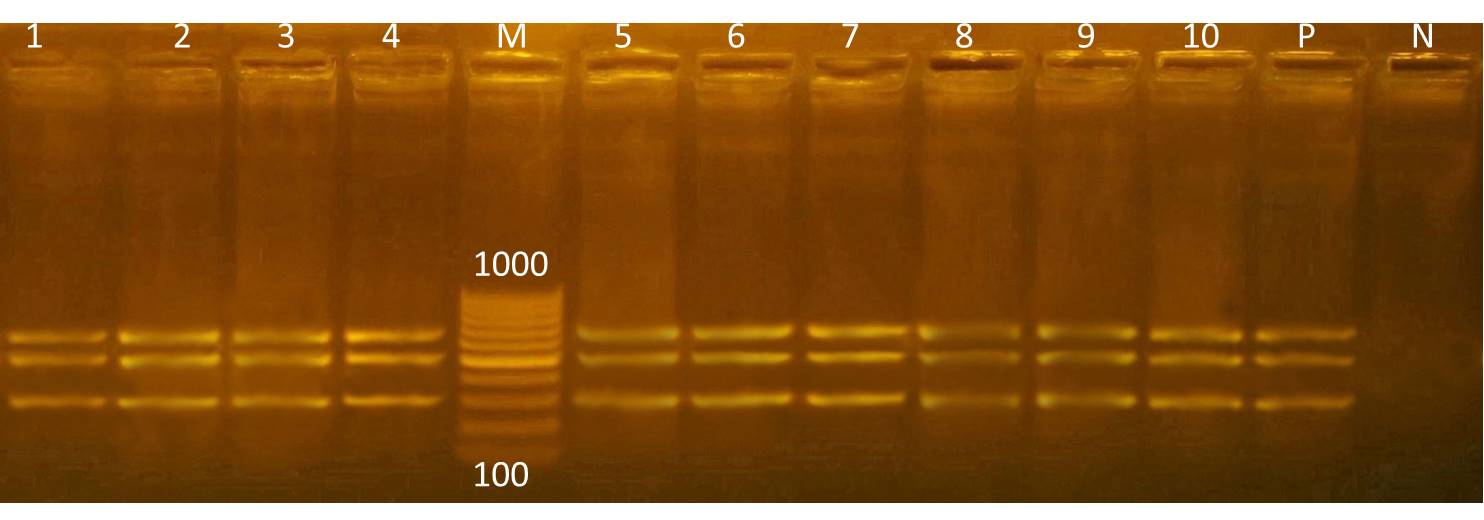

Supplement: Supplementary file 1 [file animals-11-01849-s001.zip › Figure S1.jpg]
